# Chronic Early-Life Exposure to Dicyclohexyl Phthalate (DCHP) Impairs Host Defense in *Caenorhabditis elegans* and Is Associated with Transcriptional Alterations in p38 MAPK and Insulin-like Signaling Pathways

**DOI:** 10.3390/toxics14080686

**Published:** 2026-08-03

**Authors:** Yuxuan Li, Siyuan Luo, Mingdian Lei, Lingfang Yang, Zhipeng Xie, Fengxian Liu, Lipeng Zhu, Hui Zou, Junnan Li

**Affiliations:** 1 Key Laboratory of Study and Discovery of Small Targeted Molecules of Hunan Province, School of Pharmacy, Health Science Center, Hunan Normal University, Changsha 410013, China; 2 School of Life Science and Engineering, Southwest Jiaotong University, Chengdu 611756, China; 3 School of Life Sciences, Central South University, Changsha 510006, China

**Keywords:** dicyclohexyl phthalate, innate immunity, *Caenorhabditis elegans*

## Abstract

Dicyclohexyl phthalate (DCHP) is a widely used plasticizer, but its potential impact on immunity remains largely unexplored. In this study, synchronized L1-stage *Caenorhabditis elegans* were exposed to 0.0001–0.1 g/L DCHP for 72 h to investigate the effects of chronic early-life exposure on innate immunity; 0.01 and 0.1 g/L were used for subsequent mechanistic analyses. Innate immune function was evaluated using *Pseudomonas aeruginosa* PA14 survival assays, RT-qPCR, mutant strains, and oxidative-stress-related measurements. Chronic exposure beginning at the L1 stage significantly reduced the survival of *C. elegans* infected with *Pseudomonas aeruginosa* PA14. This immunosuppression was associated with increased *daf-2* and *age-1* transcript levels and reduced *daf-16* transcript levels, indicating transcriptional alterations in insulin-like signaling-related genes. Additionally, the expression of *pmk-1*, *nsy-1*, and *sek-1*, key components of the p38 MAPK pathway, was markedly downregulated. Survival assays using different mutants supported the involvement of insulin-like signaling- and p38 MAPK-related genes in the decline in innate immunity following DCHP exposure. Furthermore, DCHP exposure reduced the expression of stress resistance genes, including *skn-1*, and several antioxidant enzymes. These findings suggest that DCHP may impair innate immune function through transcriptional alterations in immune- and stress-response pathways, although direct pathway activation or inhibition was not demonstrated.

## 1. Introduction

Phthalates are extensively used as plasticizers in a wide range of industrial and consumer products, including medical devices, cosmetics, and children’s toys [1]. The pervasive presence of phthalate-containing products in daily life raises concerns about potential health risks associated with exposure through multiple pathways, such as airborne transmission, dermal contact, and ingestion via the food chain [2]. Numerous studies have demonstrated that phthalates can induce neurotoxicity and developmental toxicity [3,4].

As endocrine-disrupting chemicals, phthalates may also exert immunomodulatory effects [5]. Research has shown that exposure to di-n-butyl phthalate (DnBP), di-ethyl phthalate (DEP), and other phthalates can alter cytokine secretion by monocytes and macrophages, and influence cellular differentiation, regeneration, and inflammatory responses in vivo [6]. In murine models, DEHP exposure disrupts immune homeostasis by reducing peripheral leukocyte counts and impairing immune function [7]. Similarly, zebrafish exposed to DBP exhibit activation of inflammatory cells and cytokines, which exacerbates hypothalamic–pituitary–adrenal (HPA) axis dysregulation and induces immune system disorders [8].

Dicyclohexyl phthalate (DCHP) is a commonly used plasticizer found in nitrocellulose, ethylcellulose, polyvinyl chloride, and various resins. Like other phthalates, DCHP readily leaches from polymers due to its weak binding affinity [9] and has been detected in human biological samples [10]. Because of potential health concerns, the U.S. Consumer Product Safety Commission prohibits DCHP concentrations above 0.1% in regulated children’s toys and child-care articles [11]. The 0.1% threshold refers to the mass fraction of DCHP in regulated product materials and is not equivalent to an environmental or biological exposure concentration. Like other plasticizers, DCHP exerts endocrine-disrupting effects. Following long-term exposure to DCHP, macrophage function in mice becomes impaired, and atherosclerosis is also induced [12]. Recent studies have reported that exposure to DCHP causes liver injury in rats due to impaired oxidative stress metabolism [13]. Furthermore, some studies have indicated that the toxic effects induced by DCHP exposure are hereditary. For instance, parental exposure to DCHP exacerbates insulin resistance and insulin signaling impairment in F1 offspring [14].

However, the immune effects of DCHP—particularly on innate immunity—remain unexplored. *Caenorhabditis elegans* provides a genetically tractable model for mechanistic studies of innate immunity because it lacks an adaptive immune system, relies on conserved immune- and stress-response pathways, and permits the use of defined mutants together with the established *Pseudomonas aeruginosa* PA14 infection assay [15,16,17]. Nevertheless, findings from this invertebrate model should be interpreted mechanistically and should not be directly extrapolated to human immunotoxicity. To address this gap, the present study exposed synchronized L1-stage nematodes to DCHP for 72 h and evaluated PA14-challenged survival, immune-related gene expression, mutant survival, antimicrobial effector expression, and oxidative stress responses. This research provides foundational insights into the immunotoxic potential of DCHP and contributes to a broader understanding of its impact on host defense mechanisms.

## 2. Materials and Methods

### 2.1. C. elegans Culture

Wild-type *Caenorhabditis elegans* N2, CB1370 (*daf-2*), TJ1052 (*age-1*), GR1307 (*daf-16*), KU25 (*pmk-1*), AU3 (*nsy-1*), KU4 (*sek-1*), CL2166 (*gst-4p::gfp*), and *Escherichia coli* OP50 were obtained from the Caenorhabditis Genetics Center (CGC). All nematodes were maintained on nematode growth medium (NGM) plates or liquid medium seeded with OP50 at 20 °C. NGM was prepared using the standard formulation per liter: 3 g NaCl, 2.5 g peptone, 20 g agar, 25 mL of 1 M potassium phosphate buffer (pH 6.0), 1 mL of 1 M CaCl_2_, 1 mL of 1 M MgSO_4_, and 1 mL of 5 mg/mL cholesterol. OP50 was cultured overnight in Luria–Bertani medium and seeded onto NGM plates before use. All maintenance of *C. elegans* was performed according to the previous reports [16,17].

### 2.2. Synchronization of Nematodes

Gravid nematodes were collected and washed three times with K medium (32 mM KCl, 51 mM NaCl). Lysis buffer (5 M NaOH: 5% NaClO = 1:2) was added, and the mixture was gently agitated until adult worms were lysed. Eggs were harvested by centrifugation at 3000 rpm and washed four times with K medium. The eggs were then placed on unseeded NGM plates and incubated for 10 h at 20 °C to obtain synchronized L1-stage larvae.

### 2.3. DCHP Exposure

The initial exposure series comprised 0.0001, 0.001, 0.01, and 0.1 g/L DCHP and was selected as a logarithmic range-finding series with reference to a previous 72-h phthalate exposure study in *Caenorhabditis elegans* [18] and the preliminary concentration-response assay in the present study. Based on the PA14-challenged survival results, 0.01 and 0.1 g/L were selected for subsequent analyses because they significantly reduced survival following infection without significantly affecting lifespan under uninfected conditions. These nominal concentrations were selected for mechanistic hazard identification rather than to simulate environmentally realistic exposure and were not derived from the 0.1% regulatory threshold for DCHP content in consumer-product materials. Synchronized L1-stage nematodes were transferred to K medium containing DCHP dissolved in dimethyl sulfoxide (DMSO); the final DMSO concentration did not exceed 0.3%, and the control group received the corresponding vehicle concentration and fed with OP50. After 72 h of incubation at 20 °C, nematodes were washed three times with K medium to remove residual OP50 and then incubated in ampicillin (100 μg/mL) for 1 h to obtain DCHP-exposed nematodes.

### 2.4. Slow-Killing Assay

DCHP-exposed nematodes were transferred to NGM plates seeded with *Pseudomonas aeruginosa* PA14. Mortality was recorded every 12 h at 20 °C. Nematodes that escaped or climbed the plate walls were excluded. Death was determined by the absence of pharyngeal pumping [19].

### 2.5. Lifespan Assay

DCHP-exposed nematodes were transferred to NGM plates seeded with OP50 at 20 °C, and the day of transfer was designated as day 0. Worms were transferred to fresh plates daily, and survival was recorded once per day. Nematodes that escaped or climbed the plate walls were excluded. Death was determined by the absence of pharyngeal pumping [19].

### 2.6. RT-qPCR Analysis

Following exposure, N2 nematodes were transferred to *P. aeruginosa* PA14-seeded NGM plates and incubated for 2 h at 20 °C. Nematodes were then harvested and snap-frozen in liquid nitrogen. Total RNA was extracted using the RNAeasy™ Animal RNA Isolation Kit with Spin Column (Beyotime Biotechnology, Cat: R0027, Shanghai, China). cDNA synthesis was performed using the iScript™ cDNA Synthesis Kit (Bio-Rad, Cat: 1708891, Hercules, CA, USA), and quantitative PCR was conducted using SYBR Green (Bio-Rad, Cat: 1725121, Hercules, CA, USA). Primer sequences are listed in Supplementary Table S1. Relative transcript levels were normalized to the reference gene *act-1* and calculated using the 2^−ΔΔCt^ method [20].

### 2.7. Measurement of Oxidative-Stress-Related Biomarkers

After exposure, N2 nematodes were transferred to *Pseudomonas aeruginosa* PA14-seeded NGM plates and incubated for 2 h at 20 °C. Nematodes were harvested and snap-frozen in liquid nitrogen. For each biological replicate, more than 2000 worms from a separately synchronized and exposed population were pooled. Protein concentration was determined using the bicinchoninic acid (BCA) assay (Beyotime, Cat: P0012, Shanghai, China). Catalase (CAT) and superoxide dismutase (SOD) activities, reduced glutathione (GSH) and malondialdehyde (MDA) content were measured using commercial kits (Beyotime, Cat: S0101S, S0051, S0053 and S0131S). CAT activity was assessed based on hydrogen peroxide decomposition, SOD activity based on inhibition of a superoxide-dependent chromogenic reaction, GSH using a colorimetric glutathione assay, and MDA using a thiobarbituric acid-reactive substances assay. Measurements were normalized to total protein content and performed according to the manufacturers’ instructions. Three independent biological replicates were analyzed.

### 2.8. Hydrogen Peroxide Resistance Assay

To assess oxidative-stress resistance, DCHP-exposed wild-type N2 worms were transferred to K medium containing 0.3 mM hydrogen peroxide (H_2_O_2_). Survival was assessed every 4 h at 20 °C under a stereomicroscope, and death was defined by the absence of movement and pharyngeal pumping. The assay was independently performed three times, with more than 60 worms in total.

### 2.9. Visualization of GST-4::GFP

Exposed CL2166 (*gst-4p::gfp*) nematodes were transferred to *Pseudomonas aeruginosa* PA14-seeded NGM plates and incubated for 2 h at 20 °C. Nematodes were then anesthetized with 40 mM sodium azide, mounted on 2% agarose pads, and observed under a fluorescence microscope (EVOS M7000, Thermo Fisher Scientific, Waltham, MA, USA). All images were acquired using identical, optimized settings (exposure time and gain constant across all groups within each replicate). Fluorescence intensity was quantified using ImageJ v1.54: whole-worm regions of interest (ROIs) were drawn (excluding the pharynx and gut granules), and mean gray values were measured. Background fluorescence (average of three cell-free areas per image) was subtracted from each worm’s value [21]. The analysis included 15 worms in total, and the experiment was independently performed three times.

### 2.10. Statistical Analysis

All data are presented as mean ± standard deviation (SD). Experiments were independently performed three times using separately synchronized, exposed, and collected worm populations, with each independent experiment considered a biological replicate. Normality was assessed using the Shapiro–Wilk test, and homogeneity of variance was assessed using the Brown-Forsythe test before analysis of variance. Survival distributions were estimated using the Kaplan–Meier method and compared using the log-rank test. Data satisfying the parametric assumptions were analyzed using one-way analysis of variance (ANOVA), followed by Dunnett’s multiple-comparisons test to compare each concentration group with the control group. Statistical analyses were performed using GraphPad Prism version 9.0 (GraphPad Software, Boston, MA, USA). All tests were two-sided, and *p* < 0.05 was considered statistically significant. Fluorescence intensity was quantified using ImageJ v1.54 software.

## 3. Results

### 3.1. DCHP Exposure Reduced the Survival of C. elegans Under P. aeruginosa PA14

**Figure 1:**
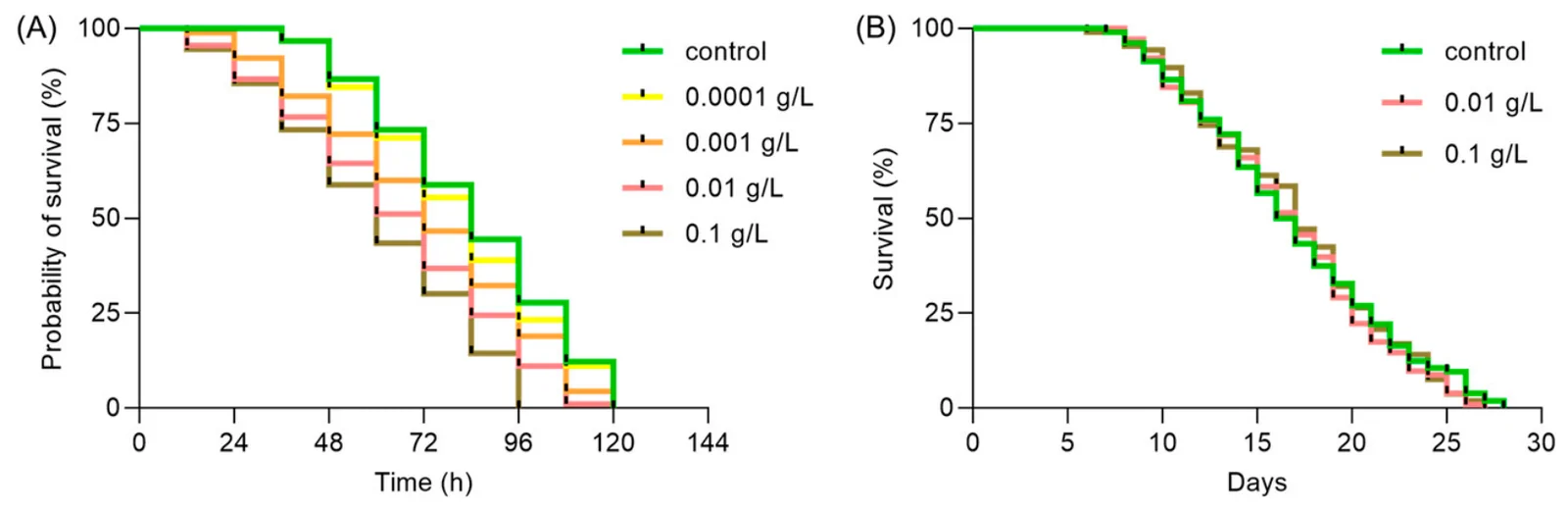
Effects of dicyclohexyl phthalate (DCHP) exposure on the survival of *Caenorhabditis elegans*. (**A**) Survival of wild-type N2 worms following *Pseudomonas aeruginosa* strain PA14 infection after exposure to DCHP for 72 h beginning at the first larval (L1) stage (*n* > 60 worms in total from three independent biological replicates). (**B**) Lifespan of N2 worms maintained on PA14-free nematode growth medium (NGM) plates after exposure to DCHP for 72 h beginning at the L1 stage (*n* > 90 worms in total from three independent biological replicates). Survival distributions were estimated using the Kaplan–Meier method and compared using the log-rank test.

The survival rate of *C. elegans* exposed to *P. aeruginosa* PA14 serves as an indicator of their innate immune capacity [17]. Long-term exposure to DCHP (0.0001 g/L) during early developmental stages did not significantly affect *C. elegans* survival compared with the control group (Figure 1A, log-rank test, *p* > 0.05). However, as the concentration of DCHP increased, a progressive decline in survival time was observed. Specifically, exposure to 0.001 g/L DCHP reduced survival from 84 h to 72.13 h (Figure 1A, log-rank test, *p* < 0.05). At 0.01 g/L, survival further decreased to 65.73 h, and at 0.1 g/L, a significant reduction to 60 h was recorded (Figure 1A, log-rank test, *p* < 0.05). These findings suggest that prolonged early-life exposure to DCHP compromises the innate immune capacity of *C. elegans*. Under baseline conditions (without PA14 infection), chronic early-life exposure to DCHP did not significantly affect the survival at 0.01 g/L and 0.1 g/L (Figure 1B, log-rank test, *p* > 0.05), suggesting that these concentrations do not cause overt baseline lethality or severe fitness reduction as measured by lifespan. However, survival assays (lifespan) are not equivalent to developmental assays, and the present study did not directly evaluate developmental parameters (e.g., body length, growth rate, brood size, or timing of developmental milestones). Therefore, we cannot exclude the possibility that early-life DCHP exposure may affect nematode development, which could in turn influence immune outcomes. These results suggest that chronic early-life exposure to DCHP compromises the ability of *C. elegans* to combat pathogenic infection, which is consistent with impaired innate immune function. The *P. aeruginosa* PA14 infection assay is a widely used model for evaluating host-defense capacity in *C. elegans*. For a toxicant like DCHP that acts in the same direction, the observed decrease in survival could theoretically involve non-specific toxicity or other physiological perturbations, even in the absence of overt baseline toxicity. Therefore, while our data support an effect on host defense, we cannot entirely exclude contributions from non-specific effects. The 0.1% regulatory threshold concerns DCHP content in regulated product materials and cannot be directly converted into an environmental or biological exposure concentration [22]. However, that regulatory limit applies to consumer product composition, not to environmental or biological exposure concentrations. In this study, the concentration of DCHP (up to 0.1 g/L) was selected for hazard identification and mechanistic toxicology experiments, rather than to mimic environmentally realistic exposure scenarios. Environmental monitoring studies have reported DCHP concentrations in aquatic systems at much lower levels than those used here, and higher concentrations are often necessary in laboratory settings to induce measurable toxic responses. Therefore, 0.01 and 0.1 g/L were selected for subsequent analyses because they reduced survival following PA14 infection without significantly altering lifespan under uninfected conditions. These nominal concentrations were used for mechanistic hazard identification rather than environmental exposure simulation.

### 3.2. Transcriptional Alterations in Insulin-like Signaling-Related Genes Following DCHP Exposure

**Figure 2:**
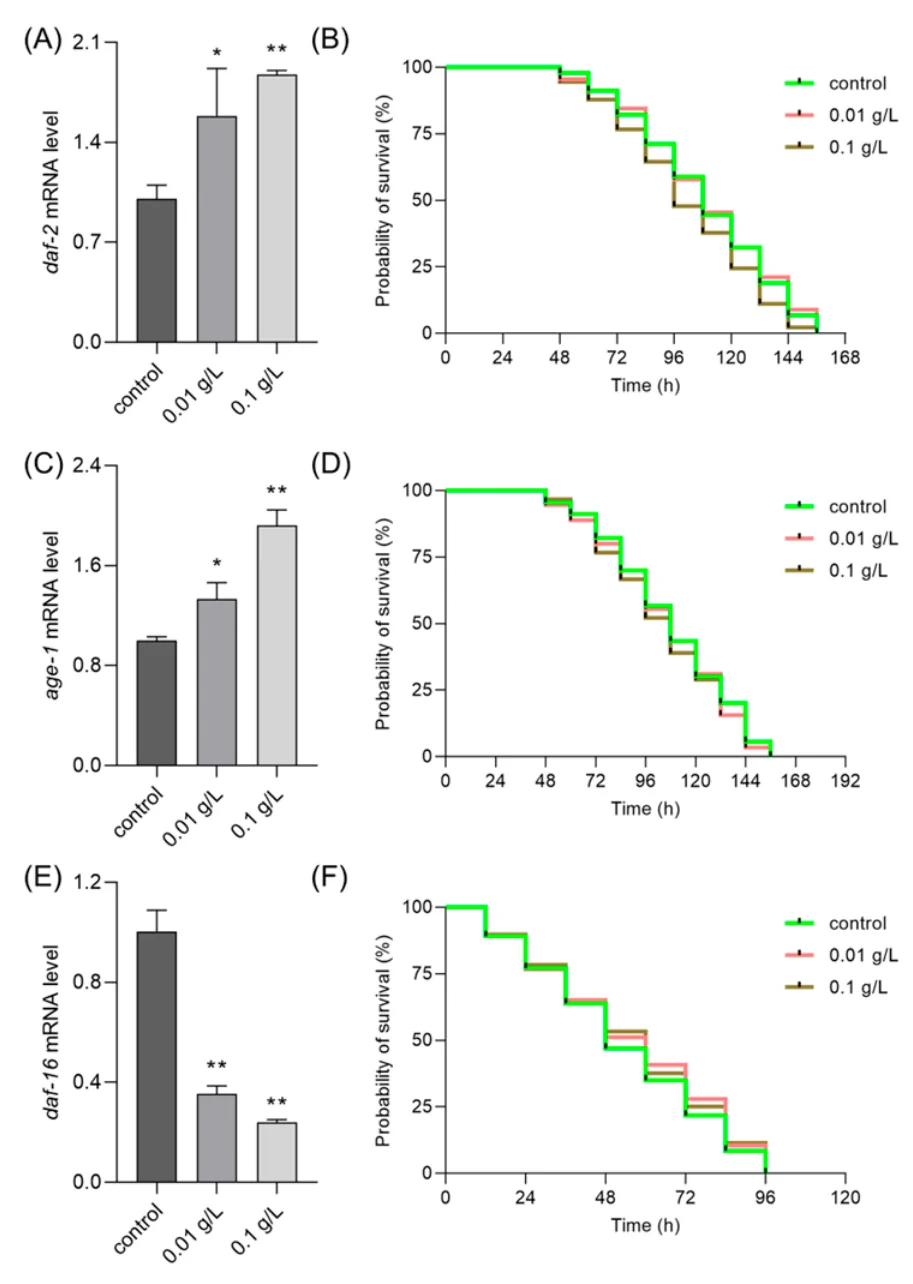
Effects of dicyclohexyl phthalate (DCHP) exposure on insulin-like signaling-related gene expression and PA14-challenged survival in corresponding mutants. Wild-type N2 worms were exposed to DCHP for 72 h beginning at the first larval (L1) stage and then infected with *Pseudomonas aeruginosa* strain PA14 for 2 h. Messenger RNA (mRNA) levels of (**A**) *daf-2*, (**C**) *age-1*, and (**E**) *daf-16* were measured using reverse transcription quantitative polymerase chain reaction (RT-qPCR; *n* > 2000 worms per group from three independent biological replicates). Survival was recorded for (**B**) CB1370 (*daf-2*), (**D**) TJ1052 (*age-1*), and (**F**) GR1307 (*daf-16*) mutants (*n* > 60 worms in total from three independent biological replicates). Survival distributions were analyzed using the Kaplan–Meier method and log-rank test. Gene-expression data were analyzed using one-way analysis of variance (ANOVA) followed by Dunnett’s multiple-comparisons test. * *p* < 0.05 and ** *p* < 0.01 versus the control group.

The insulin-like signaling pathway is known to regulate innate immunity in *C. elegans* [23]. As shown in Figure 2A, DCHP exposure significantly upregulated *daf-2* mRNA expression. Notably, DCHP did not affect the survival of *daf-2* mutants following *P. aeruginosa* PA14 infection (Figure 2B). Similarly, *age-1* mRNA expression was upregulated (Figure 2C), yet survival of *age-1* mutants remained unchanged under *P. aeruginosa* PA14 infection (Figure 2D). In contrast, *daf-16* mRNA expression was significantly reduced by early-life DCHP exposure (Figure 2E). Given that DAF-16 contributes to innate immune regulation [24], the absence of an additional DCHP-associated survival reduction in *daf-16* mutants (Figure 2F) supports the involvement of insulin-like signaling-related genes in the observed phenotype. These results indicate that DCHP exposure was associated with transcriptional changes in IIS-related genes; however, direct pathway activation was not demonstrated because DAF-16 nuclear localization, a key post-translational regulatory event, was not assessed.

### 3.3. Transcriptional Changes in the p38 MAPK Pathway Following DCHP Exposure

**Figure 3:**
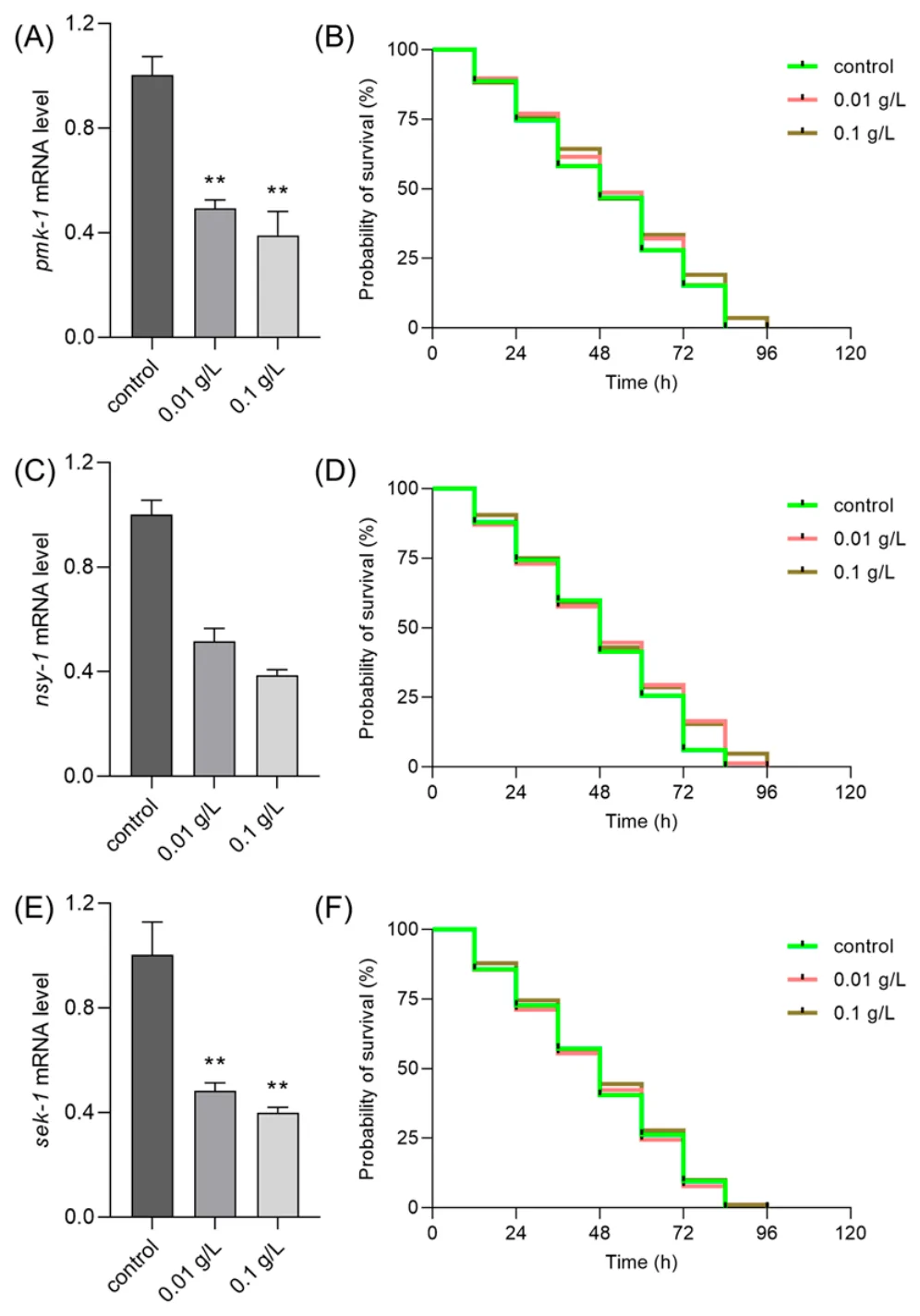
Effects of dicyclohexyl phthalate (DCHP) exposure on the transcript levels of p38 mitogen-activated protein kinase (p38 MAPK) pathway components and PA14-challenged survival in corresponding mutants. Wild-type N2 worms were exposed to DCHP for 72 h beginning at the first larval (L1) stage and then infected with *Pseudomonas aeruginosa* strain PA14 for 2 h. Messenger RNA (mRNA) levels of (**A**) *pmk-1*, (**C**) *nsy-1*, and (**E**) *sek-1* were measured using reverse transcription quantitative polymerase chain reaction (RT-qPCR; *n* > 2000 worms per group from three independent biological replicates). Survival was recorded for (**B**) KU25 (*pmk-1*), (**D**) AU3 (*nsy-1*), and (**F**) KU4 (*sek-1*) mutants (*n* > 60 worms in total from three independent biological replicates). Survival distributions were analyzed using the Kaplan–Meier method and log-rank test. Gene-expression data were analyzed using one-way analysis of variance (ANOVA) followed by Dunnett’s multiple-comparisons test. ** *p* < 0.01 versus the control group.

The p38 MAPK signaling pathway also plays a critical role in nematode innate immunity [25]. Following DCHP exposure, the expression of *pmk-1* was significantly downregulated (Figure 3A). To determine whether this transcriptional change contributes to immune dysfunction, we examined the survival of *pmk-1* mutant under *P. aeruginosa* PA14 infection. DCHP exposure did not further alter the survival of *pmk-1* mutants following *P. aeruginosa* PA14 infection (Figure 3B). Additionally, *nsy-1* expression was suppressed (Figure 3C), and survival of AU3 mutants, which lack functional *nsy-1*, was unaffected by DCHP exposure (Figure 3D), supporting the involvement of *nsy-1*. *sek-1* expression was also significantly reduced (Figure 3E), and DCHP exposure did not alter the survival of *sek-1* mutants under *P. aeruginosa* PA14 infection (Figure 3F). Thus, DCHP exposure reduced the transcript levels of several p38 MAPK pathway components, while the corresponding mutant survival assays supported the involvement of these genes in the observed phenotype. Collectively, these results indicate that impaired innate immunity following DCHP exposure was associated with transcriptional downregulation of p38 MAPK pathway components, without directly demonstrating functional inhibition of the pathway because PMK-1 phosphorylation was not assessed.

### 3.4. DCHP Reduced Antimicrobial Effector Gene Expression

**Figure 4:**
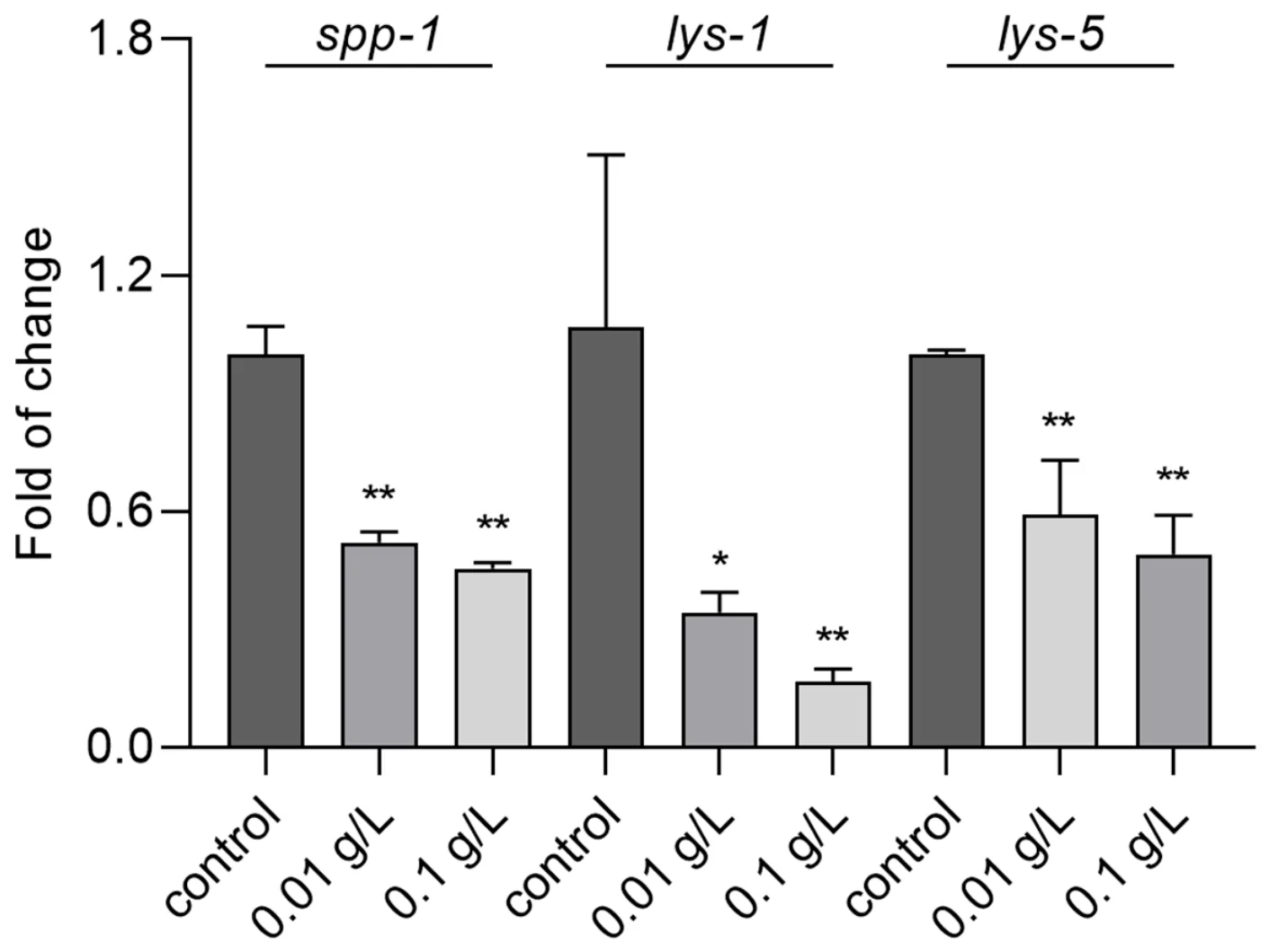
Effects of dicyclohexyl phthalate (DCHP) exposure on antimicrobial effector gene expression. Wild-type N2 worms were exposed to DCHP for 72 h beginning at the first larval (L1) stage and then infected with *Pseudomonas aeruginosa* strain PA14 for 2 h. Messenger RNA (mRNA) levels of *spp-1*, *lys-1*, and *lys-5* were measured using reverse transcription quantitative polymerase chain reaction (RT-qPCR; *n* > 2000 worms per group from three independent biological replicates). Data were analyzed using one-way analysis of variance (ANOVA) followed by Dunnett’s multiple-comparisons test. * *p* < 0.05 and ** *p* < 0.01 versus the control group.

Insulin-like signaling and the p38 MAPK pathway regulate innate immunity in *C. elegans* partly by controlling antimicrobial effector gene expression [23]. We therefore measured the transcript levels of the antimicrobial effector genes *spp-1*, *lys-1*, and *lys-5*. As shown in Figure 4, all three genes were downregulated, and these transcriptional changes were consistent with the impaired host-defense phenotype following DCHP exposure.

### 3.5. DCHP Reduced Stress-Response Capacity

**Figure 5:**
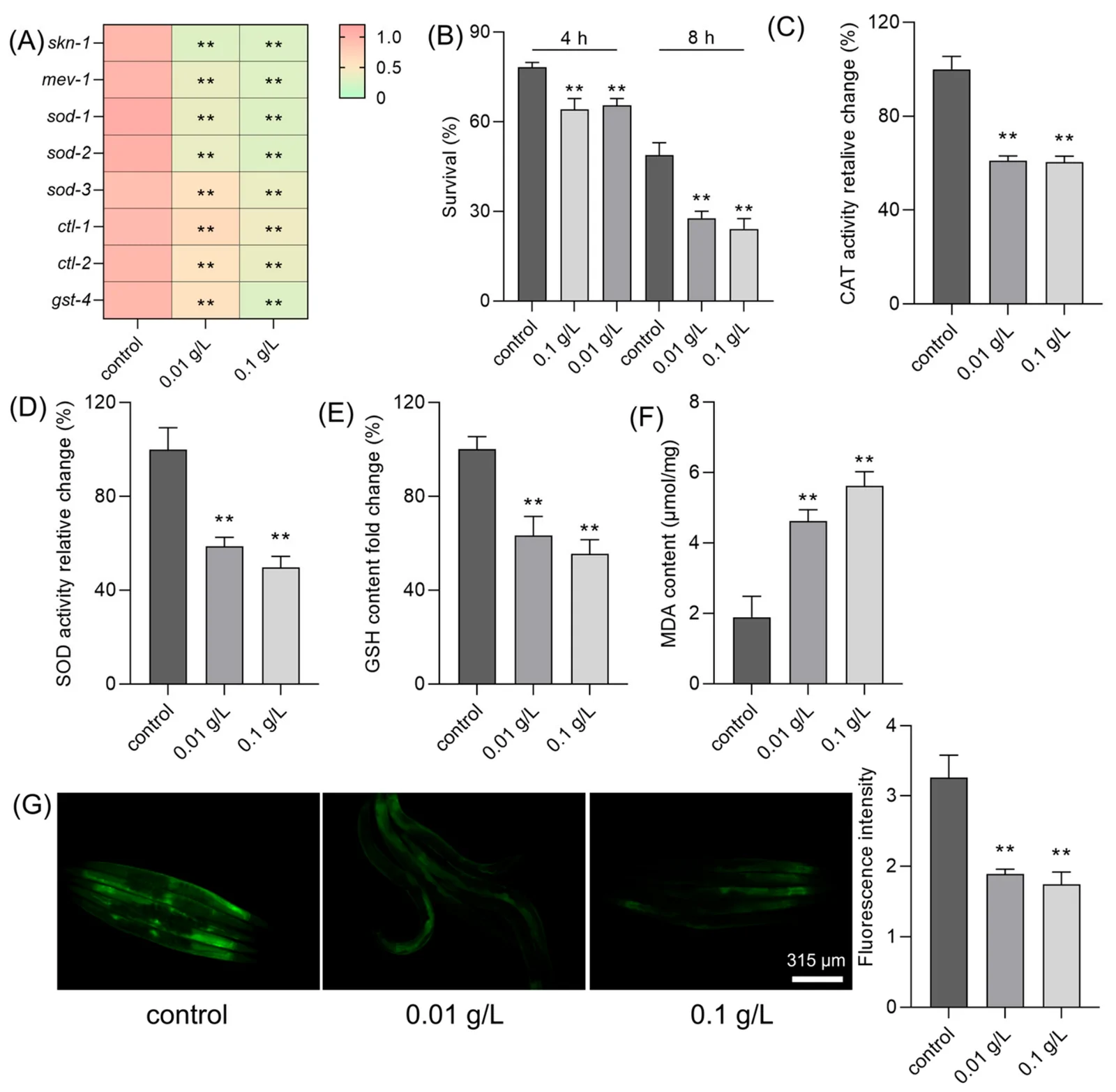
Effects of dicyclohexyl phthalate (DCHP) exposure on stress and antioxidant responses in *Caenorhabditis elegans*. Wild-type N2 worms were exposed to DCHP for 72 h beginning at the first larval (L1) stage and then infected with *Pseudomonas aeruginosa* strain PA14 for 2 h. (**A**) Transcript levels of stress-response and antioxidant-related genes were measured using reverse transcription quantitative polymerase chain reaction (RT-qPCR; *n* > 2000 worms per group from three independent biological replicates). (**B**) Survival of N2 worms was assessed after hydrogen peroxide (H_2_O_2_; 0.3 mM) challenge for 4 and 8 h (*n* > 60 worms per group). (**C**) Catalase (CAT) activity, (**D**) superoxide dismutase (SOD) activity, (**E**) reduced glutathione (GSH) content, and (**F**) malondialdehyde (MDA) content were measured in pooled worm samples (*n* > 2000 worms per group). (**G**) *gst-4p::gfp* reporter fluorescence was quantified in CL2166 worms after PA14 infection (*n* = 15 worms in total from three independent biological replicates). Data were analyzed using one-way analysis of variance (ANOVA) followed by Dunnett’s multiple-comparisons test. ** *p* < 0.01 versus the control group.

Stress response genes, including *skn-1*, and antioxidant-related genes are also implicated in the innate immune function of *C. elegans* [26]. DCHP exposure significantly downregulated *skn-1* and *mev-1* expression (Figure 5A). Moreover, genes encoding antioxidant enzymes—*sod-1*, *sod-2*, *sod-3*, *ctl-1*, *ctl-2*, and *gst-4*—were markedly suppressed. We next exposed the worms to hydrogen peroxide (H_2_O_2_) to assess oxidative-stress resistance. DCHP exposure significantly reduced survival under H_2_O_2_ challenge (Figure 5B). Exposure to 0.1 g/L DCHP resulted in a 39.61% reduction in catalase (CAT) activity (Figure 5C), while superoxide dismutase (SOD) activity decreased by 41.2% and 50.31% following exposure to 0.01 g/L and 0.1 g/L DCHP, respectively (Figure 5D). The GSH content was also reduced by 36.63% at 0.01 g/L and 44.56% at 0.1 g/L (Figure 5E). We also measured malondialdehyde (MDA) content as an indicator of lipid peroxidation in *C. elegans*. As shown in Figure 5F, MDA content was significantly increased, indicating enhanced lipid peroxidation and impaired antioxidant capacity following DCHP exposure. Next, using CL2166 nematodes, we observed that GST-4::GFP reporter fluorescence was significantly reduced in *P. aeruginosa* PA14-infected individuals exposed to DCHP during early life (Figure 5G). These findings further support the conclusion that DCHP exposure diminishes *P. aeruginosa* PA14 stress resistance and antioxidant capacity in nematodes.

## 4. Discussion

With the widespread use of plasticizers, concerns regarding their potential hazards have increasingly come to light [27]. Although most existing research has focused on the toxicity of the commonly used DEHP [28], emerging evidence suggests that DEHP exposure can compromise the innate immunity of *Caenorhabditis elegans* and accelerate immunosenescence [18]. However, whether DCHP—a structural alternative to DEHP—elicits similar immunosuppressive effects remains unclear. To address this gap, we employed a nematode model to investigate the impact of chronic early-life exposure to DCHP on innate immunity.

**Figure 6:**
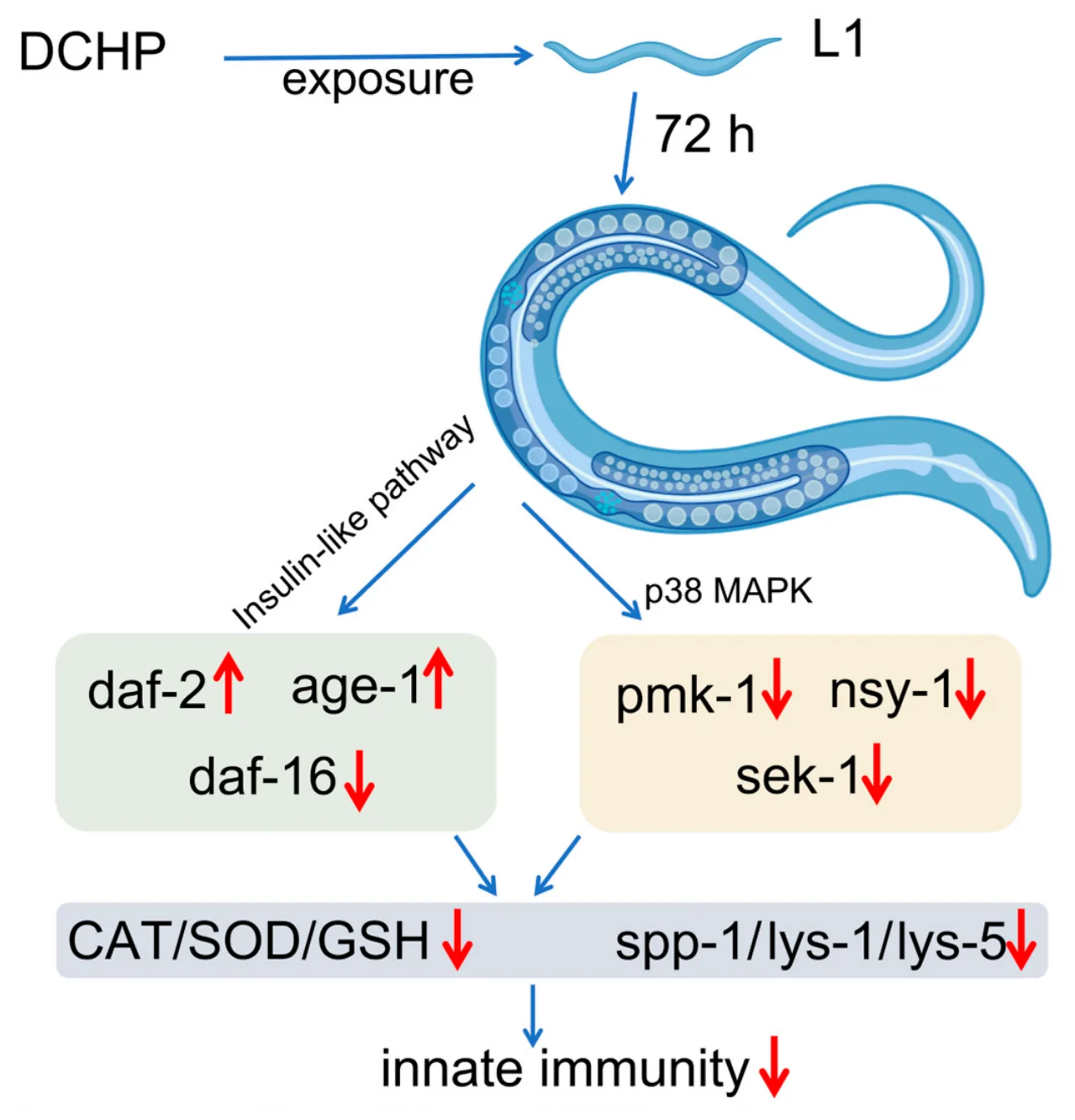
Proposed model summarizing dicyclohexyl phthalate (DCHP)-associated transcriptional and functional alterations related to innate immunity in *Caenorhabditis elegans*. The diagram summarizes experimentally observed changes and proposed relationships; the latter require further functional validation.

Our findings demonstrate that 72 H exposure to DCHP from the L1 larval stage significantly reduced the survival of nematodes challenged with *Pseudomonas aeruginosa* PA14, suggesting a diminished resistance to bacterial infection (survival assays do not directly assess development, and developmental parameters were not measured in this study). *P. aeruginosa* PA14 is known to activate *daf-2* in nematodes, thereby inhibiting the nuclear translocation of *daf-16* and modulating the insulin-like signaling pathway. However, our data show reduced *daf-16* transcription at the mRNA level. It should be noted that DAF-16 activity is primarily controlled by nuclear translocation, a post-translational event that was not directly assessed in this study. Future studies incorporating functional readouts, such as DAF-16::GFP nuclear localization assays or a broader panel of DAF-16 target genes, are needed to determine whether DCHP alters DAF-16 function. In the canonical IIS pathway, activation of DAF-2/AGE-1 signaling restricts DAF-16 nuclear localization and can reduce the expression of host-defense effectors such as *thn-2*, *lys-7*, and *spp-1*, thereby impairing innate immunity [23]. Mutations in *daf-2* and *age-1* not only extend lifespan but also enhance resistance to bacterial pathogens [29]. In our study, DCHP exposure upregulated *daf-2* and *age-1* expression at the transcriptional level, which is consistent with an alteration of the insulin-like signaling pathway, and reduced *daf-16* expression. Notably, DCHP exposure did not affect the survival of *daf-2*, *age-1*, or *daf-16* mutants under *P. aeruginosa* PA14 infection, and reduced the expression of *spp-1* (Figure 6), supporting an association between transcriptional alterations in insulin-like signaling-related genes and impaired innate immunity, while direct evidence of pathway activation based on DAF-16 nuclear localization remains to be determined.

The p38 MAPK signaling pathway plays a pivotal role in immune regulation across both animal and plant systems [30,31]. In *C. elegans*, this highly conserved pathway governs innate immune responses [25]. Upon bacterial infection, p38 MAPK promotes resistance to *P. aeruginosa* PA14 by inducing immune-related genes, including CUB-like genes [32]. In our study, DCHP exposure significantly downregulated the expression of *pmk-1*, *nsy-1*, and *sek-1*, and did not alter the survival of corresponding mutants under *P. aeruginosa* PA14 infection. These findings suggest that DCHP exposure is associated with transcriptional downregulation of p38 MAPK pathway components, which may contribute to weakened innate immunity. Additionally, the transcript levels of the antimicrobial effector genes *spp-1*, *lys-1*, and *lys-5* were reduced, consistent with the observed transcriptional changes in p38 MAPK- and insulin-like signaling-related genes (Figure 6). Although the present study demonstrates that DCHP exposure downregulates the expression of *pmk-1*, *sek-1*, and *nsy-1* in the p38 MAPK signaling pathway, with supportive evidence from the corresponding mutant survival assays, it is important to note that PMK-1 function is primarily regulated by phosphorylation. Therefore, future studies should further investigate PMK-1 phosphorylation to more directly assess the impact of DCHP exposure on its activity.

Previous studies have shown that *C. elegans* can enhance DAF-16-mediated resistance to *Enterococcus faecalis* by upregulating oxidative stress response genes such as *sod-3* and *ctl-2* [26]. Additionally, SKN-1 activation via the p38 MAPK pathway contributes to enhanced resistance [33]. In our study, DCHP exposure reduced *skn-1* expression and suppressed downstream antioxidant genes, suggesting that the observed decline in antioxidant capacity may be associated with transcriptional downregulation of p38 MAPK pathway components and altered *daf-16* expression. These findings imply that DCHP may impair stress responses in *C. elegans* by alterations in insulin-like signaling (at the transcription level) and p38 MAPK pathway gene expression, ultimately compromising innate immunity. Because transcript levels were measured only after a 2 h PA14 challenge, the present study cannot determine whether DCHP altered basal immune-gene expression, impaired PA14-induced transcriptional responses, or affected both processes. Future studies using a two-factor design comparing vehicle- and DCHP-exposed worms before and after PA14 challenge will be required to distinguish basal transcriptional effects from pathogen-induced immune responses.

Given that DAF-16 and p38 MAPK regulate antimicrobial effector genes in nematodes, the reduced transcript levels of *spp-1*, *lys-1*, and *lys-5* following DCHP exposure may contribute to the impaired host-defense phenotype. However, the present data establish associations among transcriptional and functional changes rather than a complete causal mechanism. Because *C. elegans* is an invertebrate model lacking adaptive immunity, additional studies in vertebrate models, such as zebrafish and mice, are needed to determine the broader relevance of these findings. Moreover, the DCHP concentrations used in the mechanistic experiments were substantially higher than those generally reported in environmental monitoring studies and were selected for hazard identification rather than environmental exposure simulation. In addition, although DCHP did not significantly alter lifespan under uninfected conditions, the present study did not directly assess developmental progression, feeding behavior, locomotion, or pathogen avoidance. Therefore, contributions from these physiological changes to the reduced survival following PA14 challenge cannot be completely excluded. Future studies should incorporate developmental and behavioral endpoints to distinguish immune-specific effects from broader physiological toxicity. Accordingly, this study provides mechanistic evidence of DCHP-associated immunotoxic potential under the tested conditions and supports further investigation using environmentally relevant concentrations and vertebrate models.

## 5. Conclusions

In this study, *Caenorhabditis elegans* was used to evaluate the effects of chronic early-life exposure to dicyclohexyl phthalate (DCHP) on innate immunity. Our findings revealed that DCHP exposure significantly reduced the survival of nematodes infected with *Pseudomonas aeruginosa* PA14. Mechanistic analyses showed that the impaired immune phenotype was associated with transcriptional alterations in insulin-like signaling-related genes and reduced transcript levels of p38 MAPK pathway components; mutant survival analyses supported the involvement of these genes without directly demonstrating pathway activation or inhibition. Additionally, DCHP exposure suppressed the expression of *skn-1* and reduced antioxidant capacity, which may further contribute to impaired immune responses. Collectively, these results indicate that DCHP exhibits immunotoxic potential under the tested experimental conditions. Because the exposure concentrations were selected for mechanistic hazard identification and were substantially higher than typical environmental levels, the findings should not be interpreted as a direct estimate of environmental or human health risk. This study provides foundational insights for further exploration into the immunotoxic effects of DCHP and its broader implications for environmental health.

## Data Availability

The raw data supporting the conclusions of this article will be made available by the authors on request.

## Supplementary Materials

The following supporting information can be downloaded at https://www.mdpi.com/article/10.3390/toxics14080686/s1, Table S1: The primers for q-PCR in *C. elegans*.

## Abbreviations

The following abbreviations are used in this manuscript:

**Abbreviation**	**Full Term**
CAT	Catalase
DCHP	Dicyclohexyl phthalate
DEHP	Di(2-ethylhexyl) phthalate
DMSO	Dimethyl sulfoxide
GSH	Reduced glutathione
GFP	Green fluorescent protein
HPA axis	Hypothalamic–pituitary–adrenal axis
IIS	Insulin-like signaling
MAPK	Mitogen-activated protein kinase
MDA	Malondialdehyde
NGM	Nematode growth medium
RT-qPCR	Reverse transcription quantitative polymerase chain reaction
SOD	Superoxide dismutase
SD	Standard deviation
