# Supplementary material for: Chronic Early-Life Exposure to Dicyclohexyl Phthalate (DCHP) Impairs Host Defense in Caenorhabditis elegans and Is Associated with Transcriptional Alterations in p38 MAPK and Insulin-like Signaling Pathways
Source: Toxics. 2026 Aug 3;14(8):686. doi: 10.3390/toxics14080686 (PMC13517810; doi:10.3390/toxics14080686)
Supplement: Supplementary file 1 [file toxics-14-00686-s001.zip › toxics-4451062-supplementary.pdf]

Supplementary Table S1. The primers for q-PCR in *C. elegans*.

Gene	Forward	Reverse
daf-16	TTTCCGTCCCCGAACTCA	ATTCGCCAACCCATGATGG
daf-2	GCGGATACACAGCAAGAATAAC	GAGCCACAAGCACCAGAAC
age-1	CCTGAACCGACTGCCAATC	GTGCTTGACGAGATATGTGTATTG
skn-1	AGTGTCGGCGTTCCAGATTTC	GTCGACGAATCTTGCGAATCA
sod-3	CTAAGGATGGTGGAGAACCTTCA	CGCGCTTAATAGTGTCCATCAG
gst-4	TCCGTCAATTCACTTCTTCCG	AAGAAATCATCACGGGCTGG
ctl-1	TTTCAACGGTCGCTGGAGAA	AGTCTGTGGATTGCGCTTCA
pmk-1	CGACTCCACGAGAAGGAT	ATATGTACGACGGGCATG
ctl-2	TCCCAGATGGGTACCGTCAT	GGTCCGAAGAGGCAAGTTGA
mev-1	CGTCACCAAGTCCGAGGCAAAG	ATGTGGAGCGATTGGGCGATTC
nsy-1	TGCGATGAACTACTACGG	CACCCAAATGACCAAATA
sek-1	TGCTCAACGAGCTAGACG	ATGTTCGACGGTTTCACG
sod-1	GAGTCGGAGACAAGGCAGAAGAG	AGCAGCGAGAGCAATGACACC
sod-2	TCGCTGTTCAAGGATCAGGATGG	CAAGTCCAGTTGTTGCCTCAAGTG
act-1	CCAGGAATTGCTGATCGTATGCAGAA	TGGAGAGGGAAGCGAGGATAGA
